# Bayesian prediction of bacterial growth temperature range based on genome sequences

**DOI:** 10.1186/1471-2164-13-S7-S3

**Published:** 2012-12-07

**Authors:** Dan B Jensen, Tammi C Vesth, Peter F Hallin, Anders G Pedersen, David W Ussery

**Affiliations:** 1 Technical University of Denmark, Center for Systems Biology, Denmark; 2 Novozymes A/S, Denmark

## Abstract

**Background:**

The preferred habitat of a given bacterium can provide a hint of which types of enzymes of potential industrial interest it might produce. These might include enzymes that are stable and active at very high or very low temperatures. Being able to accurately predict this based on a genomic sequence, would thus allow for an efficient and targeted search for production organisms, reducing the need for culturing experiments.

**Results:**

This study found a total of 40 protein families useful for distinction between three thermophilicity classes (thermophiles, mesophiles and psychrophiles). The predictive performance of these protein families were compared to those of 87 basic sequence features (relative use of amino acids and codons, genomic and 16S rDNA AT content and genome size). When using naïve Bayesian inference, it was possible to correctly predict the optimal temperature range with a Matthews correlation coefficient of up to 0.68. The best predictive performance was always achieved by including protein families as well as structural features, compared to either of these alone. A dedicated computer program was created to perform these predictions.

**Conclusions:**

This study shows that protein families associated with specific thermophilicity classes can provide effective input data for thermophilicity prediction, and that the naïve Bayesian approach is effective for such a task. The program created for this study is able to efficiently distinguish between thermophilic, mesophilic and psychrophilic adapted bacterial genomes.

## Background

Being able to infer from genome sequences the optimal habitat of uncultured strains, and thus infer the selectional pressures under which the organism has evolved, can save time and money. The laborious tasks of selecting and optimizing strains for production of enzymes relevant for specific industrial purposes can be time-consuming as well as expensive. Thus, being able to predict the optimal habitat conditions of a microbial organism, based solely on its genomic sequence, would be beneficial, as now it is possible to sequence bacteria that have never been cultured, and culturing for some organisms may be quite difficult, if not impossible [1].

This study aims to provide a method of predicting bacterial preferences regarding thermophilicity, *i.e*. the broad range of temperatures where the bacterium has optimal growth. From the literature, unfortunately there appear to be no single standard for how the optimal temperature (OT) range of any thermophilicity class is defined [2-5], and many authors do not explicitly define which definitions they use [6-8]. For this study, the four included classes were defined as: hyperthermophiles (OT > 80°C), thermophiles (OT 50°C-80°C), mesophiles (OT 15-50°C) and psychrophiles (OT < 15°C).

Due to the fact that Guanine/Cytosine (GC) base-pair bonds have three hydrogen bonds, compared to Adenosine/Tyrosine (AT) with only two hydrogen bonds [9], it has been suggested that a higher overall GC content might be a general adaptation to high temperatures [10]. For this reason, many have looked for such a correlation, with varying conclusions; some have reported an increase in genomic GC-content correlated with an increase in optimal growth temperature, and others have shown the same for higher GC content of coding, as well as non-coding regions of specific prokaryotic genes [3,5]. Different groups, however, have reported that no such correlation could be seen for prokaryotic DNA [2,7], and a plot of GC content vs. OT shows no clear correlation. Perhaps most interestingly, in a study where a mesophile *Escherichia coli *strain was experimentally evolved into a facultative thermophile, Blaby *et al. *reported 31 point mutations to have taken place [11]. In that evolutionary case, mutations turning an A or a T into a G or a C occurred as frequently as a G or a C being replaced by an A or a T; 41.9 percent of the time. In short, it appears that the last word may not yet have been said on this matter.

Hurst *et al. *[7] find a possible correlation between temperature and higher GC-content in the structural RNA and the more freely evolving third position of the codons of coding sequences [7]. For protein coding genes, if the third codon position does correlate in such a way, then codon usage information might very well be useful in relation to optimal temperature prediction. In addition, Smole *et al. *[4] report a very impressive performance of predictive distinction between mesophile and thermophile organisms based on proteomic amino acid features, and Gromiha *et al. *[12] demonstrated high accuracy in discriminating mesophile from thermophile proteins, based on amino acid composition. According to the literature on the subject, the only attempt at adapting a predictive strategy for psychrophiles, as well as meso- and thermophiles, includes just a single psychrophile data point [13].

Although prediction attempts have previously been based on proteomes derived from fully sequenced genomes, so far there are no published attempts to include the presence or absence of protein families associated with thermophilicity classes in the predictions. This study aims to do this, while comparing such predictions with corresponding predictions based on AT/GC-count (genomic and 16S rDNA), codon- and amino acid usage. Further, this study will evaluate the predictive performance achieved by basing the predictions on thermophilicity-class correlated protein families in combination with the mentioned structural features.

In the context of organism-centered thermophilicity prediction, as opposed to protein-centered predictions, previous studies have implemented such strategies as linear regression, neural networks and random forest [13,4]. This study implements a naïve Bayesian classifier (see Method section for details). This method has, in spite of its relative simplicity, proven an effective predictive tool in a range of fields, including taxonomic classification [14], prediction of genetic risk factors [15,16], and discrimination of mesophile and thermophile proteins, based on amino acid composition [12]. As an additional advantage, unlike the case of black-box optimization methods, such as artificial neural networks etc., the trained parameters of the naïve Bayesian classifier directly shows how much of an influence, a given parameter has on a given feature.

## Results and discussion

### Phylogenetic relationships of included genomes

This study aims to predict the optimal thermophilicity range of bacteria based on genomic information. To this end, a training set of 9 hyperthermophiles (OT > 80°C), 40 thermophiles (OT 50°C-80°C), 28 mesophiles (OT 15-50°C) and 11 psychrophiles (OT < 15°C) were found (before training set reduction, see Method section). The predictions were tested on a test set of 6 hyperthermophiles, 9 thermophiles 7 mesophiles and 7 psychrophiles.

**Figure 1:**
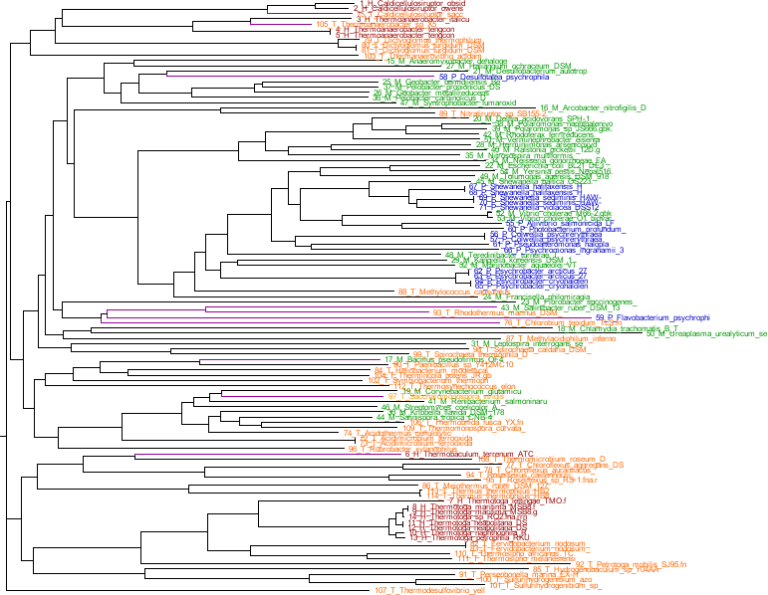
**Phylogenetic relationships of 117 bacteria from the four different thermophilicity classes**. The relationship is based on predicted 16S rRNA sequences. Red tips are hyperthermophiles, orange are thermophiles, green are mesophiles and blue are psychrophiles. The purple lines indicate the species exemplifying evolutionary flexibility, as discussed in the text.

Examining the phylogenetic relationship of these 117 genomes (training and test sets) offers a hint of the evolutionary flexibility, associated with these thermophilicity classes. Figure 1 shows this relationship in the form of a neighbor-joining tree from predicted 16S rDNA sequences. The tips are color coded to indicate the thermophilicity classes; red is hyperthermophile, orange is thermophile, green is mesophile and blue is psychrophile.

All hyperthermophiles are found in clades consisting only of hyperthermophiles and thermophiles, indicating that a thermophile ancestry may well be required before obtaining hyperthermophilicity. Some hyperthermophiles appear to have evolved from thermophile ancestors, but some strains, such as *Thermoanaerobacter *sp X5, appear to be thermophile versions of otherwise hyperthermophile genera, and are as such more likely to have evolved their thermophilicity from a hyperthermophile ancestor.

Some thermophiles appear to have evolved from mesophiles, such as the case of *Saccharomonospora viridis*. However, the reverse transition is also possible, as is seen for *Bacillus pseudofirmus *OF4. Some psychrophiles, such as *Desulfotalea psychrophila *appear to have evolved from mesophiles, but no mesophiles appear to be obvious candidates for psychrophile descendants. The thermophile *Chlorobium tepidum *TLS is equally related at another thermophile (*Rhodothermus marinus *DSM), a mesophile (*Salinibacter ruber *DSM) as well as a psychrophile (*Flavobacterium psychrophi*).

The mentioned organisms, exemplifying evolutional flexibility, are marked in Figure 1 by their purple, rather than black, branch lines. A rooted tree showing the bootstrap values is visible in Additional file 1, and the.dnd-file from which the tree is based is found as Additional file 2.

In the making of the tree, multiple sequence alignment was done using Clustalw [17] and optimized by employing the bootstrapping algorithm [18], using the standard value of 1000 bootstraps. The vast majority of the nodes have bootstrapping values in the range of 900 to 1000, although three nodes had values in the single-digit range and six nodes had values in the two-digit range. The predicted 16S rDNA sequences of three different archaea (*Acidianus hospitalis *W1, *Desulfurococcus kamchatkensis 1221n *and *Caldivirga maquilingensis *IC-167) were included to provide an obvious rooting point.

### Class associated protein families

To allow the prediction of thermophilicity class based on protein family content, overrepresented protein families were determined for each class. A protein family was considered overrepresented in a given class, if it was found to be present in more than 65% of the members of that class (in the training set), and found only at a significantly lower rate in all other classes (p < 0.01). The number of protein families found to be overrepresented in each class is given in Table 1:

**Table 1:** The number of protein families found to be overrepresented in each of the three thermophilicity classes.

Class of overrepresentation	Number of protein families
Thermophiles	8
Mesophiles	0
Psychrophiles	32

Overrepresentation is defined as presence in more than 65% of one class, and at a significantly (p < 0.01) lower frequency in all other classes.

The sequences of the members of the class-associated protein families are given in Additional file 3 and their likelihood given the three classes are seen in Additional file 4.

Notice that no protein families are found to be overrepresented in the mesophile genomes. This could conceivably be caused by the fact that the mesophile portion of the training set (28 genomes, 24 genera) was relatively large compared to the psychrophile (8 genomes, 7 genera) and the thermophile (13 genomes, 9 genera). However, another explanation could be that the mesophiles as a class are simply more diverse and less specialized than the other classes. This makes sense, given the vast range of different temperate habitats that a mesophile bacterium could inhabit. This hypothesis would be supported, if the described tendency of hot- and cold-associated protein families, but few to no medium-temperature associated proteins families, continued to be found when access to larger datasets, including specific optimal growth temperatures for each genome, becomes available.

### Predictive performance

To assess the effectiveness of sequence features and class-associated protein families as the basis of Bayesian prediction, predictions were performed based on only sequence features, only protein families and a combination of the two.

Table 2 shows the predictive performance of the naïve Bayesian inference, based on an assumed Gaussian distribution of the observed sequence features, the overrepresented protein families and the combination of those two datasets. The predictive performance is evaluated using a Matthews correlation coefficient (MCC) for predictions of each of the classes individually (shown in Additional files 5, 6 and 7). By this evaluation, a value of 0 indicates a random guess, 1 indicates a perfect correlation between prediction and actual class and -1 indicates a perfect anti-correlation.

**Table 2:** The predictive performance of the naïve Bayesian inference program, achieved when implementing a Gaussian likelihood function of a.) the observed structural characteristics alone, b.) when implementing the observed protein family frequencies alone as likelihoods and c.) when combining the observed protein family frequencies with the Gaussian likelihood functions of observed structural characteristics.

Class	Test set	
**a. Structural features**	**MCC**	**% Correct predictions**

Thermophiles	0.24	80.0
Mesophiles	0.36	50.0
Psychrophiles	0.47	25.0

**b. Protein families**	**MCC**	**% Correct predictions**

Thermophiles	0.60	92.9
Mesophiles	0.13	28.6
Psychrophiles	0.51	50.0

**c. Combined**	**MCC**	**% Correct predictions**

Thermophiles	0.67	92.0
Mesophiles	0.40	57.1
Psychrophiles	0.68	50.0

(For the individual predictions, see Additional file 5, 6 and 7)

To put the obtained MCC values into perspective, the percentage of correct predictions for each class is shown. For a simple Boolean question (*e.g*. psychrophile - yes or no), the fraction of correct predictions in a series of random guesses would be 50%; for three possible answers (thermophile, mesophile or psychrophile), the corresponding fraction would be 33%, assuming a balanced test set. However, the due to the nature of the data, the test set is not balanced; given the composition of the test set (see Method section), a random guess of a thermophile would be correct 56% of the time, mesophile 28% of the time and psychrophile 16% of the time. As can be seen from Table 2, all of the predictions are better than what would be expected from random guessing.

The predictive performance, as indicated by the MCC value, from prediction of mesophile genomes is relatively low when predictions are based on protein family data alone, compared to sequence features alone. In fact, the percentage of mesophiles correctly predicted given protein family data is slightly better than random guessing. However, the MCC values for predictions of thermophiles and psychrophiles are well above what is expected from random chance, as are the corresponding percentages of correct predictions.

This shows protein family data to contain more information in relation to psychrophile and thermophile adaptations, compared with mesophile adaptations, which is consistent with the finding of protein family over-representation.

Noticing the relatively low MCC for thermophile predictions, when predictions are based entirely on sequence features, one might suspect the variability of these features to be higher in thermophiles compared to the two other classes. When comparing the variance in the amino acid usage from the three thermophilicity classes, obtained from the training set (see Additional file 8), it is seen that 60% of the amino acids have the highest variance in the thermophile class, with the remaining 40% of the amino acids being most variably used in mesophiles. If one looks at the variance in the use of codons, however, the situation is nearly the exact reverse, with 62.5% of the codons being most variable in the mesophile class and 37.5% of the codons having the highest variance within the thermophiles. This, given the lower accuracy of prediction of thermophiles using sequence features, indicates that the amino acid usage profile of a bacterial genome holds more information than codon usage profiles, in terms of thermophilicity class prediction in general. This is also reflected by the high hit rates for thermophiles (80%) accompanied by the low MCC-value of 0.24, indicating a high tendency to predict thermophiles, regardless of the actual class.

For prediction of all thermophilicity classes, an increased accuracy is clearly seen when combining the sequence features and the protein family information, bringing the predictive performance for all three classes well above random levels. This indicates that both types of data in their own right carry useful information about bacterial thermal adaptation.

It is worth noticing that the lowest MCC value is still found for prediction of mesophiles. If one looks at the predictions (Additional file 7), it is seen that half of all erroneous predictions are mesophiles being predicted as thermophiles, and the other half are thermophiles and psychrophiles being predicted to be mesophiles.

The two psychrophiles predicted to be mesophiles were *Flavobacterium psychrophilum *JIP02 86 and *Psychrobacter cryohalolentis *K5. *F. psychrophilum *JIP02 86 has an optimal growth temperature at 15°C [21], which puts it right at the defining edge between mesophiles and psychrophiles, and *P. cryohalolentis *K5 is a psychrophile known to grow at any temperature between -10°C and 30°C [22]. It is thus not surprising that some commonalities between these two psychrophiles and the broad mesophile dataset have been found, making these two genomes appear mesophile to the predictor.

As previously mentioned the mesophile dataset spans a vast range of temperatures and vary greatly in habitat. This combined with the above discussed variability in sequence features of thermophiles and mesophiles would account for the three erroneous predictions of thermophiles as mesophiles.

We expect such errors to be reduced when the method is applied to a larger data set, including more specific values for optimal temperature, which would allow the training of the method for more fine-grained predictions. In the meantime, we wish to once again point to the fact that all predictions are better than random, demonstrating the usefulness of the method.

### Conditional feature independence

The basic premise of the naïve Bayesian classifier is that all features included in the classification are mutually independent. A discussion of the employed datasets in this light is thus in order.

#### Overrepresented protein families

The protein families, included as features for predictions, were selected for their overrepresentation in one of the three thermophilicity classes, as described in the Methods section. All protein families found to be overrepresented in *e.g*. the thermophile bacteria will thus be observed frequently in this particular thermophilicity class, and significantly less frequently in other classes. Thus a pattern would be expected to emerge of apparent correlations between protein families associated with the same thermophilicity class, and anti-correlations between proteins families associated with different thermophilicity classes.

**Figure 2:**
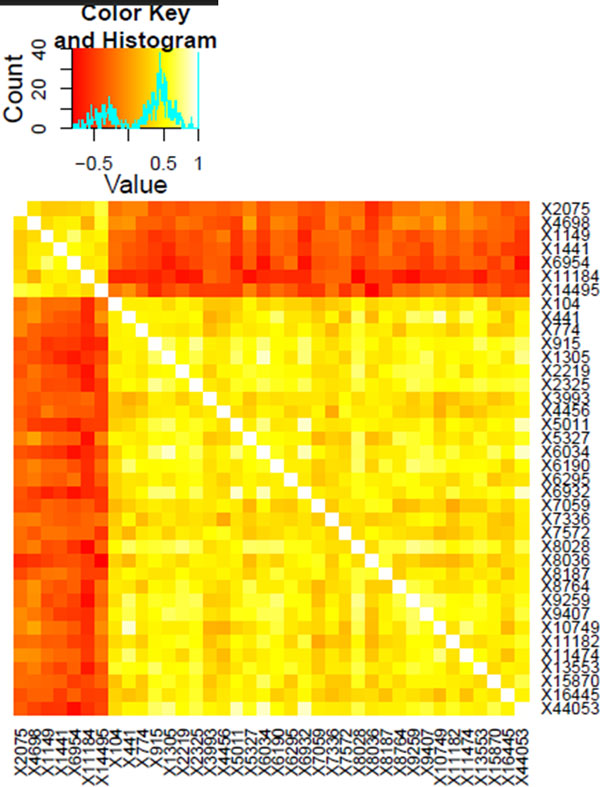
**Pearson's correlation coefficients between thermophilicity class-associated protein families, shown as a heat map**. Lighter colors indicate stronger correlations. The top seven protein families (2075, 4698, 1149, 6954, 11184 and 14495) were all found to be overrepresented in thermophile genomes. The remaining protein families were overrepresented in psychrophile genomes. Families associated with the same thermophilicity class tend to correlate moderately with each other and anti-correlate moderately with families associated with other classes.

The results of a Pearson's correlation coefficient analysis in the form of a heat map, including all protein families found to be overrepresented in one of the thermophilicity classes, is shown in Figure 2.

The pattern found in Figure 2 is as would be expected from the data. Therefore, the apparent correlations and anti-correlations in the sets of overrepresented protein families can reasonably be attributed to external selective pressures from the environment, rather than internal dependencies between the genes coding for these protein families. Granting this, the selected protein families live up the basic premise of the naïve Bayesian classifier.

#### Sequence features

As discussed in the Background section, previous studies have either shown, or made a likely case for the possibility, that specific amino acids or codons are used more frequently in thermophile compared to mesophile organisms. Some sequence feature correlation would thus be expected from external selectional pressures, similarly to the case for class-associated protein families, as discussed above.

This being said, the values of the two main categories of sequence features included in this study (amino acid and codon usage) are calculated in percentage of the complete amino acid and codon usage, respectively, and the respective values of the two categories must thus sum to 100. This fact alone means that all sequence features cannot be conditionally independent from all other; if one amino acid is used to a high extent in a given genome, some other amino acid must be used to a lesser extent, relatively speaking. In addition, all of the amino acids are of course encoded by one or more of the codons, and thus we can expect a causal correlation between codon usage and amino acid usage.

However, as showed by Domingos & Pazzani [19] the naïve Bayesian classifier can deliver excellent classification performance, even when the assumption of independence is violated. Further, H. Zhang [20] has shown mathematically that even strong dependencies between features would not affect the naïve Bayesian classification, when those dependencies cancel each other out.

**Figure 3:**
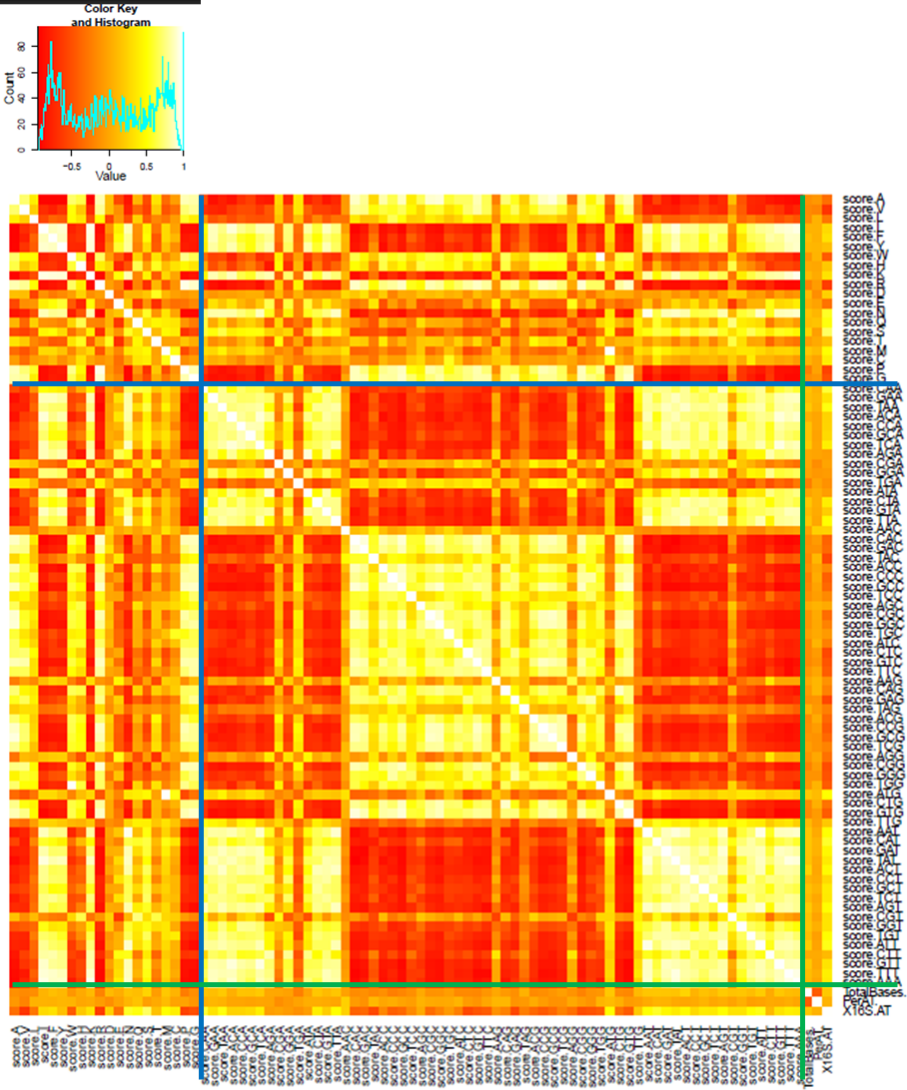
**Pearson's correlation coefficients of sequence features, presented as a heat map**. Lighter color indicates stronger correlation. The blue lines separate the amino acids from the codons, while the green lines separate the codons from the genome size and the AT content (genomic and 16S rDNA).

The results of a Pearson's correlation coefficient analysis between all sequence features, shown as a heat map, are shown on Figure 3.

From visually inspecting the heat map in Figure 3, patterns of both correlations and anti-correlations are clearly seen. If one focuses on the correlations between amino acids (upper left corner, separated from the correlations between codons by the blue lines), both extremes are seen, but near-zero values (orange) appear most common. Conversely, for correlations between codons (big square, framed by the blue and the green lines), the values near any of the two extremes are most, and approximately equally, common. Interestingly, the genomic AT count does not correlate with the AT content of 16S rDNA (small square, bottom right corner), illustrating the sensibility in including both of these two as independent features.

Lastly, a calculation of the mean of all correlations in the in the matrix yields a value of 0.008. We thus argue that the 87 features can indeed form a meaningful basis for a naïve Bayesian classifier.

## Methods

### Selection of genomes for training and test set

Given that the purpose of this study was to predict bacterial optimal growth temperature range based on published genomes, it was considered necessary to ensure the accuracy of the temperature annotations of the genomes used in the training set. To this end, the completed genomes, publicly available via the National Center for Biotechnology Information (NCBI), were cross-referenced with experimental data for optimum temperature [23].

As of the 16^th ^of March 2012, a total of 1596 completed genomes were available from NCBI [24]. Of these, 1240 came with temperature annotations. These were 20 hyperthermophiles, 91 thermophiles, 1112 mesophiles and 17 psychrophiles. To ensure a well-documented training set, the temperature range annotation of the published genomes were cross-referenced with experimental data for optimum growth temperature for 636 bacterial strains [23]. By this method, 9 hyperthermophiles, 37 thermophiles, 78 mesophiles and 11 psychrophiles had been specifically shown to have an optimal temperature within the previously described range of their annotated thermophilicity class (hyperthermophiles: OT > 80°C, thermophiles: OT 50°C-80°C, mesophiles: OT 15-50°C, psychrophiles: OT < 15°C). For six strains, the optimal growth temperature was not found to conform to the range of the annotated class. For the remaining strains, the experimental data did not specifically identify the optimum growth temperature, but rather the growth temperature used to prepare the bacteria.

The 78 confirmed mesophiles included strains representing the entire mesophile temperature spectrum, as defined above. A set of 29 genomes, spanning the mesophile spectrum in a similar fashion, was selected to be included in the training set. A total of 37 thermophiles were selected, as well as all 9 confirmed hyperthermophiles and all the 11 confirmed psychrophiles were used. The number of selected mesophiles was reduced in the interest of saving computational time.

The strains selected for the training set, along with their thermophilicity classification, is seen in Additional file 9.

A number of genomes, found not to be of the same species as any genome in the previously defined training set, were downloaded from the NCBI web page. These were 6 hyperthermophiles (3 genera), 9 thermophiles (8 genera), 7 mesophiles (7 genera) and 7 psychrophiles (4 genera).

The strains selected for the test set, along with their thermophilicity classification, is seen in Additional file 10.

Due to the low number of available hyperthermophiles genera, the hyperthermophiles were joined together with the thermophiles for training predictions.

To further reduce the overlap between the genomes in the training set and test set, all genomes found to share thermophilicity class and genus with any genome in the test set were removed from the training set.

In the end, the training set consisted of 28 mesophiles (24 genera), 13 (hyper) thermophiles (9 genera) and 8 psychrophiles (7 genera).

### Extraction of protein families

Genes were predicted for each genome in the training set, with the purpose of enabling the inclusion of thermophilicity class associated protein families into the prediction of optimum temperature. All predicted genes of all analyzed genomes were subsequently organized into families listed in a single file, thus yielding the pan-genome of the training set.

Predicted open reading frames (ORF) were determined using Prodigal (**Pro**karyotic **Dy**namic Programming **G**enefinding **Al**gorithm) [25], and the DNA- and protein-coded FASTA sequences of the predicted genes were extracted. The predicted genes were subsequently divided into protein families. This was achieved by first renaming the protein-coded FASTA-files of each genome using the MD5 algorithm [26], to ensure all genes having specific, unique names. Afterwards, the protein-BLAST algorithm [27] was used to organize the genes of each of the genomes into family clusters; for each of the genomes, a basic local alignment search test (BLAST) was performed between all of the predicted genes using BLASTp version 2.0 with default settings [28]. If a BLAST-hit was found to show an identity score of more than 50 percent in more than 50 percent of the length of the longest gene, the two genes were considered to be part of the same family. Any additional genes found to meet these criteria for at least one member of a given established family, was considered to be a member of that family. This single linkage clustering approach means that on occasion, some smaller families would merge into larger ones [29]. This will in some cases mean that individual genes, considered being in the same family, are potentially far apart in terms of similarity.

When all genomes are included in the analysis described above, the result is a group file, representing the pan-genome for the entire training set, organized into protein families, with each line corresponding to a protein family, followed by the MD5 coded names of all genes present in the family. Based on the lines of this pan-genome group file genomes in which an individual gene was located, were identified, and the genome name added to the line of the group file.

### Identification of temperature-correlated protein families

Based on the modified pan-genome group file described above, an analysis of which protein families correlate with specific thermophilicity classes could be done. Knowing which genomes belongs to which temperature class, it was possible to identify which protein families were overrepresented in each of the classes. In the present study, a protein family was said to be overrepresented in one class, if it met two criteria: first, to be overrepresented in a given class, the family should be observed in more than 65 percent of the genomes belonging to that class. Secondly, the difference between the highest class-specific frequency and the remaining three class-specific frequencies should be statistically significant (p < 0.01). If a protein family met these criteria, the number designating that family was written to a file, along with the observed frequencies of that family in the four different temperature classes, and which class it was found to be over-represented in. This file (hence forth called the likelihood file) thus designates the observed likelihoods of the selected protein families, given each class of thermophilicity.

The content of the likelihood file is sorted by the class in which it was overrepresented (*i.e*. the fourth column of the file). Next, the members of the protein families selected to be included in the likelihoods file were identified. The FASTA sequences for each member of every selected family were extracted.

### Prediction of optimum temperature of test set genomes

Based on the observed features of the different thermophilicity classes test set genomes could be predicted to belong to one of the four classes, based on naïve Bayesian inference. The general Bayesian approach estimates posterior probabilities of a given genome to belong to any of the four thermophilicity classes based on an assumption of prior probability ("background probability") of each class and the specific observations made for the specific genome, following Bayes theorem:

$$p\left(\mathrm{M|}E\right)=\frac{p\left(\mathrm{E|}M\right)\cdot p\left(M\right)}{p\left(E\right)}$$

Or:

$$posterior\;probability=\frac{likelihood\;of\;event\;E\;given\;model\cdot prior\;probability\;of\;the\;model}{total\;probability}$$

In this equation, the model *M *is a given thermophilicity class (thermophile, mesophile or psychrophile), the event *E *is a specific observation made for the genome (*e.g*. the presence or absence of a given protein family), and the total probability is the sum of the numerators of the four different probability calculations.

In the naïve Bayesian approach, all observed events are assumed to be mutually independent. This means that to update the posterior probability of any given model, one simply multiply the numerator of the relevant probability calculation with the likelihood for the observation given the model.

This approach means that the total probability is not calculated until all relevant observations have been taken into account. In the present study, the prior probability of each class was evenly divided (*i.e*. 33.33% initial probability of each).

### Predictions based on protein families

To ascertain which of the thermophilicity classes a test set of genomes belong to, it is necessary to investigate which of the predicted protein families are present in those genomes. To determine the presence/absence of protein families found to be overrepresented to specific thermophilicity classes, a protein-BLAST was performed with an expectation value of 10^-5^. Here each genome in the test set was defined as a database, and all sequences from the selected protein families were used as queries. The output was written to an xml-file. The individual BLAST-hits were considered significant if they could show a similarity of more than 50 percent in more than 50 percent of the longest sequence. If a genome could meet these criteria for all BLAST-included members of a given protein family, that family was considered to be present in the genome. This produced a tabulator-separated presence/absence (1/0) matrix, with each row representing a genome (database in the BLAST), and each column representing a protein family.

Based on this matrix and the previously described likelihood file, the thermophilicity prediction was done for each row, corresponding to each of the test set genomes. The four likelihoods of the presence of these protein families are given by a line in the likelihood file, starting with the number of the protein family, the likelihoods of which it describes. If a given protein family is found to be present in a given genome, the probability of that genome belonging to any of the four groups is updated by a factor of the observed likelihood for each of those groups, p(family|class). If the protein family is found not to be present, the probability is updated by a factor of 1- p(family|class).

A pseudo-count of 0.1 was used for all likelihoods. The python script used to perform the prediction-related calculations is seen in Additional file 11.

### Extraction of sequence features

Basic sequence features were retrieved from the genomes, to form a separate basis for predictions, to which the performance of gene family based predictions could be compared.

From the gene FASTA file for each genome of the training set, the scores were calculated for codon-, and amino acid usage (percent) as well as the genome size (number of base pairs) and relative use of AT in the protein-coding DNA as well as the sequences predicted to code for 16S ribosomal RNA (16S rDNA). Thus for each genome, a score was found for the use of the 64 different possible codons and the 20 different biologically active amino acids. These, combined with genome size and the two values of AT count, yielded a total of 87 structural features.

### Predictions based on sequence features

For each genome in the test set, all observed sequence features (codon- and amino acid usage, genome size, genomic and 16S rDNA AT count) were found. Based on these numbers, the mean (*μ_c_*) and standard deviation $\sigma_{c}^{2}$ were calculated for the corresponding numbers found for the individual groups, *c_i _*in the training set, where *i *is a number from 1 to 87, representing one of the 87 included sequence features. A Gaussian probability of the genome belonging to each of the four groups was calculated as such:

$$P\left(M|c_{i}\right)=\frac{1}{\sqrt{2\pi \sigma_{c_{i}}^{2}}}\cdot e^{-\frac{{\left(\nu -\mu_{c_{i}}\right)}^{2}}{2\sigma_{c_{i}}^{2}}}$$

For each of the 87 features, the probability of the genome belonging to each of the three groups, *M*, is updated by a factor of *P*(*M*|*c_i_*). The python script used to perform these calculations is provided in Additional file 12.

### Predictions based on sequence features and protein family data combined

The two prediction methods were combined by using the posterior probabilities based on sequence feature-based predictions as prior probabilities in predictions based on protein family presence. Expressed as a single equation, this combination looks as follows:

$$p\left(\mathrm{M|}E\right)=\frac{p\left(E|M\right)\cdot \prod_{1}^{87}\left(\frac{1}{\sqrt{2\pi \sigma_{c_{i}}^{2}}}\cdot e^{-\frac{{\left(\nu -\mu_{c_{i}}\right)}^{2}}{2\sigma_{c_{i}}^{2}}}\right)}{p\left(E\right)}$$

The python script used for this purpose is provided in Additional file 13.

### Evaluation of predictive performance

The predictions were evaluated using Matthews correlation coefficient [30]. To achieve this, the predictor was evaluated on the predictions for each class individually by considering the three classes as two classes; the one the genome belongs to, and the one the genome does not belong to. The python script used to calculate the evaluations are provided in Additional file 14.

## Conclusions

This study shows that the presence or absence of specific protein families can be used to enhance the prediction of thermophilicity classes of bacteria, based on genomic sequences, beyond what is achieved by prediction on AT/GC content (genomic and 16S rDNA), codon usage and amino acid usage. This enhanced effect can be achieved, regardless of whether the biological function of the included protein families is known or not. It is further demonstrated that prediction of a bacteria being psychrophile is possible with a fair degree of accuracy, a possibility that to our knowledge has not previously been clearly demonstrated. Lastly, this study demonstrates that the implementation of naïve Bayesian inference is effective in predicting bacterial thermophilicity class, adding this approach to the list of neural networks, random forest and linear regression analysis, which have previously been shown in the literature to be useful in this respect.

The protein families found to be significant in terms of thermophilicity prediction were agnostically selected based on trained parameters. These trained parameters thus provide a practical starting point for the natural next step of investigating the function of these families in order to form a mechanistic model for how the observed adaptations are achieved.

## List of abbreviations used

16S rDNA: the DNA sequences coding for 16S ribosomal RNA; AT/GC content: the fraction of Adenosine and Tyrosine (AT) or Guanine/Cytosine (GC) of a DNA sequence; BLAST: Basic local alignment search test; MCC: Matthews correlation coefficient; ORF: open reading frame; OT: Optimal temperature.

## Competing interests

PFH is employed at the company Novozymes A/S, the main focus of which is that of discovery and production of enzymes.

## Authors' contributions

Conceived the project: PFH, DU. Designed analytical procedures: DBJ, TCV, AGP. Contributed genome selection criteria: DU. Wrote the paper: DBJ. Manuscript critique: TCV, DU, AGP, PFH. All authors read and approved the final manuscript.

## Supplementary Material

Additional File 1 A rooted tree showing the bootstrap values (*.pdf). Click here for file

Additional File 2 **16s tree dendrogram colourcoded for all genomes (NEXUS tree file; can be viewed with a phylogenetic tree viewer such as TreeView)**. Click here for file

Additional File 3 The sequences of the members of the class-associated protein families (*.txt). Click here for file

Additional File 4 The likelihood of the members of the class-associated protein families (*.txt). Click here for file

Additional File 5 The predictive performance of the naïve Bayesian inference program, achieved when implementing a Gaussian likelihood function of the observed structural characteristics (*.txt). Click here for file

Additional File 6 The predictive performance of the naïve Bayesian inference program, achieved when implementing the observed protein family frequencies alone as likelihoods (*.txt). Click here for file

Additional File 7 The predictive performance of the naïve Bayesian inference program, achieved when combining the observed protein family frequencies with the Gaussian likelihood functions of observed structural characteristics (*.txt). Click here for file

Additional File 8 Mean and standard deviations of structural features of the training set, used as the basis for predictions, assuming a Gaussian distribution of the features. (*.doc). Click here for file

Additional File 9 The strains selected for the training set, along with their thermophilicity classification. (*.txt). Click here for file

Additional File 10 The strains selected for the test set, along with their thermophilicity classification. (*.txt). Click here for file

Additional File 11 The python script used to perform the prediction-related calculations (*.py, can be read with any text viewer). Click here for file

Additional File 12 The python script used to calculate the probability of the genome belonging to each of the three groups (*.py, can be read with any text viewer). Click here for file

Additional File 13 The python script used in the combined prediction, using the posterior probabilities based on sequence feature-based predictions as prior probabilities in predictions based on protein family presence (*.py, can be read with any text viewer). Click here for file

Additional File 14 The python script used to evaluate the predictions for each class individually by considering the three classes as two classes; the one the genome belongs to, and the one the genome does not belong to. (*.py, can be read with any text viewer). Click here for file
