# Supplementary material for: Bayesian prediction of bacterial growth temperature range based on genome sequences
Source: BMC Genomics. 2012 Dec 7;13(Suppl 7):S3. doi: 10.1186/1471-2164-13-S7-S3 (PMC3521210; doi:10.1186/1471-2164-13-S7-S3)
Supplement: Additional File 8 — Mean and standard deviations of structural features of the training set, used as the basis for predictions, assuming a Gaussian distribution of the features. (*.doc). [file 1471-2164-13-S7-S3-S8.docx]

Mean and standard deviations of structural features of the training set, used as the basis for predictions, assuming a Gaussian distribution of the features.

Table S1: Amino acid usage of the four thermophilicity classes (hyperthermophiles = H, thermophiles = T, mesophiles = M and psycrophiles = P), expressed
as means and standard deviations (st.dv)

|  |  | score.G | score.A | score.V | score.L | score.I | score.F | score.Y | score.W | score.H | score.K | score.R | score.D | score.E | score.N | score.Q | score.S | score.T | score.M | score.C | score.P |
| --- | --- | --- | --- | --- | --- | --- | --- | --- | --- | --- | --- | --- | --- | --- | --- | --- | --- | --- | --- | --- | --- |
| H | Mean | 6,956 | 6,361 | 8,292 | 10,069 | 7,435 | 4,846 | 3,595 | 1,114 | 1,630 | 7,207 | 5,402 | 4,998 | 8,289 | 3,682 | 2,327 | 5,770 | 4,609 | 2,316 | 0,758 | 4,028 |
|  | St.dv | 0,152 | 1,119 | 0,279 | 0,124 | 0,839 | 0,475 | 0,047 | 0,050 | 0,039 | 2,340 | 0,585 | 0,006 | 0,977 | 0,210 | 0,309 | 0,171 | 0,024 | 0,006 | 0,011 | 0,250 |
| T | Mean | 3,266 | 1,151 | 3,517 | 0,106 | 1,102 | 0,889 | 1,655 | 0,775 | 1,139 | 0,397 | 1,692 | 0,140 | 2,099 | 0,533 | 1,256 | 1,490 | 1,641 | 1,053 | 2,429 | 1,655 |
|  | St.dv | 9,522 | 0,629 | 3,802 | 0,004 | 0,844 | 0,318 | 0,777 | 0,434 | 1,408 | 0,121 | 0,933 | 0,003 | 5,088 | 0,141 | 1,106 | 2,623 | 0,181 | 0,344 | 1,962 | 0,267 |
| M | Mean | 7,573 | 10,081 | 7,236 | 10,454 | 5,582 | 3,909 | 2,730 | 1,234 | 2,145 | 4,477 | 6,007 | 5,412 | 6,113 | 3,495 | 3,861 | 6,077 | 5,277 | 2,310 | 1,052 | 4,655 |
|  | St.dv | 1,070 | 5,674 | 0,492 | 0,273 | 1,901 | 0,392 | 0,273 | 0,074 | 0,038 | 2,938 | 2,056 | 0,272 | 0,406 | 1,796 | 0,545 | 0,581 | 0,169 | 0,130 | 0,065 | 0,543 |
| P | Mean | 6,702 | 8,582 | 6,657 | 10,334 | 6,847 | 4,096 | 3,079 | 1,160 | 2,176 | 5,599 | 4,281 | 5,553 | 5,944 | 4,536 | 4,434 | 6,742 | 5,555 | 2,565 | 1,068 | 3,778 |
|  | St.dv | 0,136 | 0,271 | 0,015 | 0,065 | 0,100 | 0,063 | 0,005 | 0,006 | 0,009 | 0,153 | 0,113 | 0,077 | 0,098 | 0,153 | 0,101 | 0,050 | 0,050 | 0,024 | 0,018 | 0,024 |

Table S2: the usage of codons 1 though 19 in the four thermophilicity classes, expressed as means and st.dv

|  |  | score  AAA | score. CAA | score. GAA | score. TAA | score. ACA | score. CCA | score. GCA | score. TCA | score. AGA | score. CGA | score. GGA | score. TGA | score. ATA | score. CTA | score. GTA | score. TTA | score. AAC | score. CAC | score. GAC |
| --- | --- | --- | --- | --- | --- | --- | --- | --- | --- | --- | --- | --- | --- | --- | --- | --- | --- | --- | --- | --- |
| H | Mean | 4,197 | 0,727 | 5,048 | 0,082 | 1,403 | 1,054 | 1,769 | 0,873 | 2,574 | 0,311 | 2,875 | 0,186 | 3,194 | 0,504 | 1,396 | 0,835 | 2,167 | 0,954 | 2,029 |
|  | St.dv | 2,219 | 0,141 | 1,617 | 0,002 | 0,132 | 0,041 | 0,169 | 0,098 | 0,258 | 0,010 | 0,363 | 0,004 | 0,561 | 0,187 | 0,463 | 0,752 | 0,279 | 0,051 | 0,141 |
| T | Mean | 1,864 | 1,446 | 3,450 | 0,956 | 1,092 | 1,856 | 2,477 | 0,463 | 2,375 | 2,413 | 2,015 | 1,840 | 2,090 | 1,799 | 3,538 | 0,068 | 1,144 | 1,607 | 2,430 |
|  | St.dv | 1,287 | 1,518 | 7,224 | 0,427 | 0,164 | 2,810 | 2,925 | 0,063 | 1,421 | 4,175 | 2,107 | 0,813 | 0,584 | 0,836 | 3,179 | 0,001 | 0,595 | 1,681 | 3,726 |
| M | Mean | 2,392 | 1,490 | 3,199 | 0,112 | 0,745 | 0,732 | 1,587 | 0,691 | 0,393 | 0,501 | 1,082 | 0,142 | 0,537 | 0,483 | 0,830 | 1,167 | 1,913 | 1,187 | 2,795 |
|  | St.dv | 3,512 | 1,104 | 1,845 | 0,004 | 0,301 | 0,210 | 0,426 | 0,244 | 0,147 | 0,048 | 0,466 | 0,009 | 0,138 | 0,143 | 0,221 | 2,597 | 0,121 | 0,157 | 1,700 |
| P | Mean | 3,868 | 2,782 | 3,646 | 0,201 | 1,319 | 1,242 | 2,507 | 1,388 | 0,571 | 0,514 | 0,870 | 0,042 | 1,272 | 1,209 | 1,622 | 3,308 | 1,694 | 0,713 | 1,518 |
|  | St.dv | 0,625 | 0,441 | 0,375 | 0,001 | 0,065 | 0,066 | 0,103 | 0,062 | 0,041 | 0,026 | 0,093 | 0,000 | 0,102 | 0,120 | 0,175 | 1,581 | 0,050 | 0,020 | 0,047 |

Table S3: the usage of codons 20 though 38 in the four thermophilicity classes, expressed as means and st.dv

|  |  | score. TAC | score. ACC | score. CCC | score. GCC | Score .TCC | score. AGC | Score. CGC | score. GGC | score. TGC | score. ATC | score. CTC | score. GTC | score. TTC | score. AAG | score. CAG | score. GAG | score. TAG | score. ACG | score. CCG |
| --- | --- | --- | --- | --- | --- | --- | --- | --- | --- | --- | --- | --- | --- | --- | --- | --- | --- | --- | --- | --- |
| H | Mean | 2,230 | 1,180 | 0,969 | 1,525 | 1,063 | 0,985 | 0,351 | 1,115 | 0,348 | 2,487 | 2,486 | 1,440 | 2,610 | 3,010 | 1,601 | 3,241 | 0,047 | 1,063 | 0,865 |
|  | St.dv | 0,289 | 0,130 | 0,133 | 0,335 | 0,106 | 0,079 | 0,144 | 0,379 | 0,010 | 0,716 | 1,107 | 0,121 | 0,949 | 0,331 | 0,127 | 0,520 | 0,001 | 0,104 | 0,049 |
| T | Mean | 0,860 | 0,765 | 1,567 | 1,699 | 1,214 | 1,979 | 2,999 | 2,605 | 1,368 | 1,716 | 0,791 | 2,633 | 1,590 | 0,771 | 0,932 | 1,465 | 0,848 | 0,771 | 0,625 |
|  | St.dv | 0,405 | 0,370 | 2,271 | 1,598 | 0,108 | 0,125 | 6,145 | 2,701 | 0,388 | 2,070 | 0,140 | 1,957 | 1,347 | 0,353 | 0,360 | 0,537 | 0,572 | 0,254 | 0,316 |
| M | Mean | 1,476 | 2,422 | 1,330 | 3,981 | 1,220 | 1,501 | 2,372 | 3,457 | 0,677 | 2,868 | 2,070 | 2,060 | 2,025 | 2,085 | 2,371 | 2,914 | 0,065 | 1,293 | 1,819 |
|  | St.dv | 0,164 | 0,699 | 0,434 | 3,757 | 0,258 | 0,234 | 1,931 | 2,542 | 0,077 | 0,551 | 1,510 | 0,704 | 0,396 | 0,648 | 0,538 | 1,838 | 0,001 | 0,263 | 0,827 |
| P | Mean | 1,030 | 1,744 | 0,576 | 2,033 | 0,460 | 1,366 | 1,018 | 2,070 | 0,389 | 2,005 | 0,904 | 1,204 | 1,032 | 1,731 | 1,652 | 2,299 | 0,068 | 0,982 | 0,677 |
|  | St.dv | 0,046 | 0,146 | 0,095 | 0,595 | 0,116 | 0,064 | 0,080 | 0,123 | 0,005 | 0,404 | 0,339 | 0,241 | 0,130 | 0,276 | 0,251 | 0,418 | 0,000 | 0,057 | 0,021 |

Table S4: the usage of codons 39 though 57 in the four thermophilicity classes, expressed as means and st.dv

|  |  | score. GCG | score. TCG | score. AGG | Score .CGG | Score .GGG | score. TGG | score. ATG | score. CTG | score. GTG | score. TTG | score. AAT | score. CAT | score. GAT | score. TAT | score. ACT | score. CCT | score. GCT | score. TCT | score. AGT |
| --- | --- | --- | --- | --- | --- | --- | --- | --- | --- | --- | --- | --- | --- | --- | --- | --- | --- | --- | --- | --- |
| H | Mean | 1,336 | 0,705 | 1,609 | 0,236 | 1,085 | 1,114 | 2,316 | 2,388 | 2,947 | 1,447 | 1,515 | 0,676 | 2,969 | 1,365 | 0,963 | 1,140 | 1,732 | 1,296 | 0,849 |
|  | St.dv | 0,146 | 0,038 | 0,134 | 0,042 | 0,134 | 0,050 | 0,006 | 0,710 | 0,585 | 0,129 | 0,740 | 0,043 | 0,128 | 0,410 | 0,143 | 0,084 | 0,306 | 0,033 | 0,016 |
| T | Mean | 1,628 | 0,364 | 2,116 | 1,848 | 1,668 | 2,273 | 7,497 | 9,000 | 7,543 | 10,651 | 6,589 | 4,113 | 3,245 | 1,214 | 1,844 | 5,356 | 6,349 | 5,062 | 7,056 |
|  | St.dv | 0,518 | 0,044 | 2,663 | 1,462 | 1,348 | 2,996 | 1,629 | 9,831 | 0,993 | 1,544 | 6,547 | 0,943 | 0,842 | 0,108 | 0,129 | 11,555 | 5,028 | 0,301 | 1,102 |
| M | Mean | 2,985 | 1,127 | 0,390 | 1,274 | 1,354 | 1,234 | 2,310 | 3,950 | 2,879 | 1,532 | 1,582 | 0,958 | 2,618 | 1,254 | 0,818 | 0,774 | 1,528 | 0,794 | 0,744 |
|  | St.dv | 2,857 | 0,182 | 0,113 | 0,940 | 0,520 | 0,074 | 0,130 | 3,863 | 1,099 | 0,454 | 1,919 | 0,165 | 1,318 | 0,602 | 0,318 | 0,218 | 0,660 | 0,576 | 0,222 |
| P | Mean | 1,646 | 0,669 | 0,241 | 0,249 | 0,770 | 1,160 | 2,565 | 1,495 | 1,530 | 1,590 | 2,842 | 1,463 | 4,036 | 2,049 | 1,510 | 1,283 | 2,395 | 1,310 | 1,549 |
|  | St.dv | 0,117 | 0,020 | 0,033 | 0,031 | 0,039 | 0,006 | 0,024 | 0,452 | 0,058 | 0,163 | 0,190 | 0,026 | 0,067 | 0,045 | 0,077 | 0,017 | 0,108 | 0,053 | 0,045 |

Table S5: the usage of codons 58 though 64 in the four thermophilicity classes, expressed as means and st.dv

|  |  | score. CGT | score. GGT | score. TGT | score. ATT | score. CTT | score. GTT | score. TTT |
| --- | --- | --- | --- | --- | --- | --- | --- | --- |
| H | Mean | 0,320 | 1,881 | 0,411 | 1,754 | 2,410 | 2,508 | 2,236 |
|  | St.dv | 0,018 | 0,052 | 0,003 | 0,829 | 0,168 | 0,377 | 0,715 |
| T | Mean | 3,357 | 2,951 | 5,300 | 4,880 | 1,979 | 0,827 | 4,874 |
|  | St.dv | 2,088 | 0,583 | 0,598 | 0,483 | 0,125 | 0,044 | 1,444 |
| M | Mean | 1,078 | 1,679 | 0,375 | 2,177 | 1,252 | 1,466 | 1,884 |
|  | St.dv | 0,274 | 0,624 | 0,052 | 2,431 | 0,475 | 0,665 | 1,153 |
| P | Mean | 1,689 | 2,993 | 0,680 | 3,570 | 1,827 | 2,300 | 3,064 |
|  | St.dv | 0,096 | 0,071 | 0,010 | 0,378 | 0,162 | 0,147 | 0,195 |

Table S6 - The means and standard deviations of the total genomic

|  |  | TotalBases: | PerAT: | 16srRNA-Per.AT: |
| --- | --- | --- | --- | --- |
| H | Mean | 2584675 | 54,659 | 37,506 |
|  | St.dv | 2,E+12 | 25,771 | 3,760 |
| T | Mean | 2849900 | 48,366 | 40,294 |
|  | St.dv | 3,E+12 | 243,529 | 9,883 |
| M | Mean | 4226367 | 44,972 | 45,143 |
|  | St.dvi | 7,E+12 | 111,050 | 6,422 |
| P | Mean | 3764613 | 56,761 | 46,888 |
|  | St.dv | 1,E+12 | 9,424 | 0,511 |
