# Supplementary material for: Bayesian prediction of bacterial growth temperature range based on genome sequences
Source: BMC Genomics. 2012 Dec 7;13(Suppl 7):S3. doi: 10.1186/1471-2164-13-S7-S3 (PMC3521210; doi:10.1186/1471-2164-13-S7-S3)
Supplement: Additional File 1 — A rooted tree showing the bootstrap values (*.pdf). [file 1471-2164-13-S7-S3-S1.pdf]

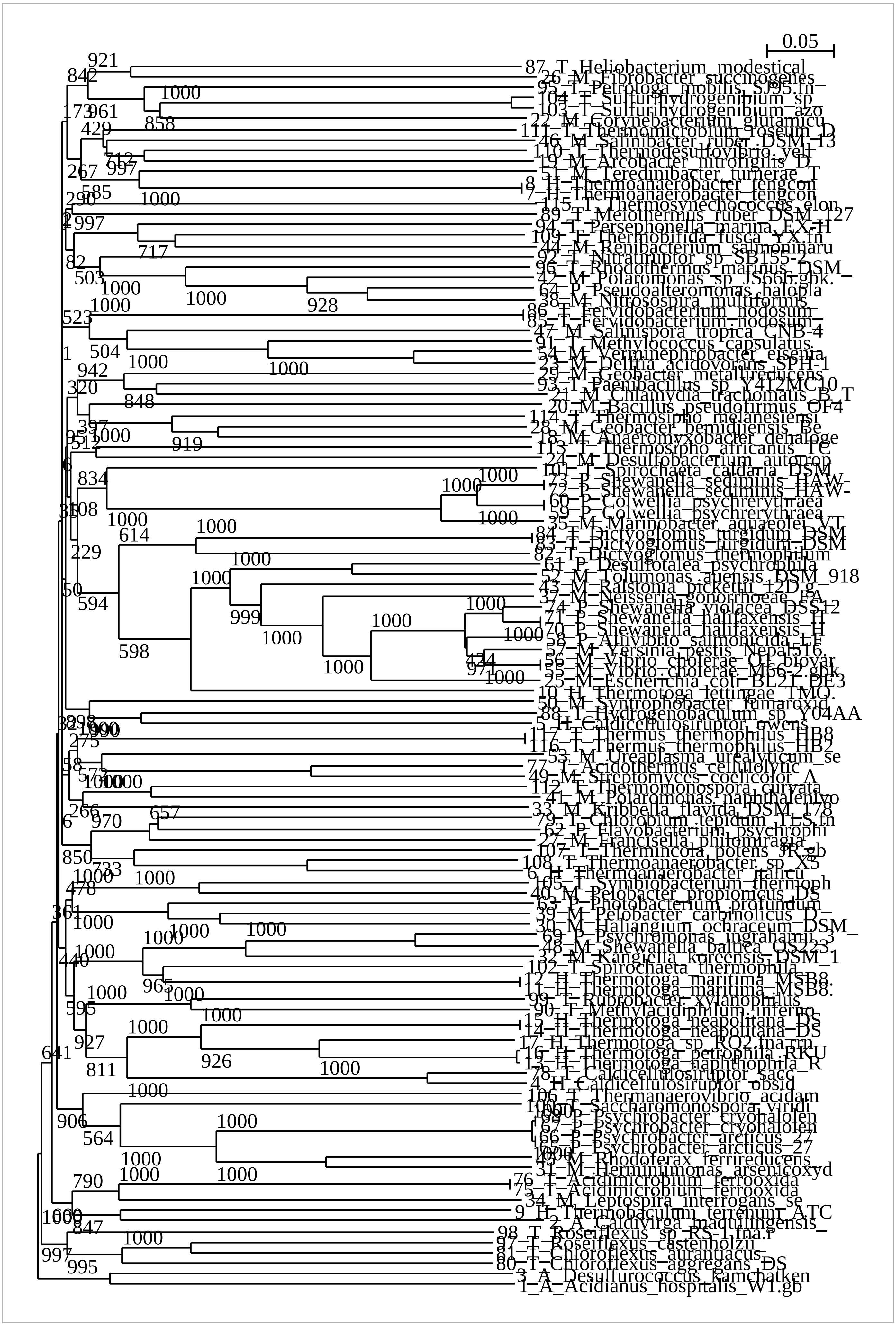
0.05
87_T_Heliobacterium_modestical
26_M_Fibrobacter_succinogenes
95_T_Petrotoga_mobilis_SJ95.fn
104_T_Sulfurihydrogenibium_sp
103_T_Sulfurihydrogenibium_azo
22_M_Corynebacterium_glutamicu
111_T_Thermomicrobium_roseum_D
46_M_Salinibacter_ruber_DSM_13
110_T_Thermodesulfovibrio_yell
19_M_Arcobacter_nitrofigilis_D
51_M_Teredinibacter_turnerae_T
8_H_Thermoanaerobacter_tengcon
7_H_Thermoanaerobacter_tengcon
115_T_Thermosynechococcus_elon
89_T_Meiothermus_ruber_DSM_127
94_T_Persephonella_marina_EX-H
109_T_Thermobifida_fusca_YX.fn
44_M_Renibacterium_salmoninaru
92_T_Nitratiruptor_sp_SB155-2
96_T_Rhodothermus_marinus_DSM
42_M_Polaromonas_sp_JS666.gbk.
64_P_Pseudoalteromonas_halopla
38_M_Nitrosospira_multiformis
86_T_Fervidobacterium_nodosum
85_T_Fervidobacterium_nodosum
47_M_Salinispora_tropica_CNB-4
91_T_Methylococcus_capsulatus
54_M_Verminephrobacter_eiseniae
23_M_Delftia_acidovorans_SPH-1
29_M_Geobacter_metallireducens
93_T_Paenibacillus_sp_Y412MC10
21_M_Chlamydia_trachomatis_B_T
20_M_Bacillus_pseudofirmus_OF4
114_T_Thermosipho_melanesiensi
28_M_Geobacter_bemidjiensis_Be
18_M_Anaeromyxobacter_dehaloge
113_T_Thermosipho_africanus_TC
24_M_Desulfobacterium_autotrop
101_T_Spirochaeta_caldaria_DSM
73_P_Shewanella_sediminis_HAW-
72_P_Shewanella_sediminis_HAW-
60_P_Colwellia_psychrerythraea
59_P_Colwellia_psychrerythraea
35_M_Marinobacter_aquaeolei_VT
84_T_Dictyoglomus_turgidum_DSM
83_T_Dictyoglomus_turgidum_DSM
82_T_Dictyoglomus_thermophilum
61_P_Desulfotalea_psychrophila
52_M_Tolumonas_auensis_DSM_918
43_M_Ralstonia_pickettii_12D.g
37_M_Neisseria_gonorrhoeae_FA
74_P_Shewanella_violacea_DSS12
71_P_Shewanella_halifaxensis_H
70_P_Shewanella_halifaxensis_H
58_P_Aliivibrio_salmonicida_LF
57_M_Yersinia_pestis_Nepal516.
56_M_Vibrio_cholerae_O1_biovar
55_M_Vibrio_cholerae_M66-2.gbk
25_M_Escherichia_coli_BL21_DE3
10_H_Thermotoga_lettingae_TMO.
50_M_Syntrophobacter_fumaroxid
88_T_Hydrogenobaculum_sp_Y04AA
5_H_Caldicellulosiruptor_owens
117_T_Thermus_thermophilus_HB8
116_T_Thermus_thermophilus_HB2
53_M_Ureaplasma_urealyticum_se
77_T_Acidothermus_cellulolytic
49_M_Streptomyces_coelicolor_A
112_T_Thermomonospora_curvata
41_M_Polaromonas_naphthalenivo
33_M_Kribbella_flavida_DSM_178
79_T_Chlorobium_tepidum_TLS.fn
62_P_Flavobacterium_psychrophi
27_M_Francisella_philomiragia
107_T_Thermincola_potens_JR.gb
108_T_Thermoanaerobacter_sp_X5
6_H_Thermoanaerobacter_italicu
105_T_Symbiobacterium_thermoph
40_M_Pelobacter_propionicus_DS
63_P_Photobacterium_profundum
39_M_Pelobacter_carbinolicus_D
30_M_Haliangium_ochraceum_DSM
69_P_Psychromonas_ingrahamii_3
48_M_Shewanella_baltica_OS223
32_M_Kangiella_koreensis_DSM_1
102_T_Spirochaeta_thermophila
12_H_Thermotoga_maritima_MSB8.
11_H_Thermotoga_maritima_MSB8.
99_T_Rubrobacter_xylanophilus
90_T_Methylacidiphilum_inferno
15_H_Thermotoga_neapolitana_DS
14_H_Thermotoga_neapolitana_DS
17_H_Thermotoga_sp_RQ2.fna.rrn
16_H_Thermotoga_petrophila_RKU
13_H_Thermotoga_naphthophila_R
78_T_Caldicellulosiruptor_sacc
4_H_Caldicellulosiruptor_obsid
106_T_Thermanaerovibrio_acidam
100_T_Saccharomonospora_viridi
68_P_Psychrobacter_cryohalolen
67_P_Psychrobacter_cryohalolen
66_P_Psychrobacter_arcticus_27
65_P_Psychrobacter_arcticus_27
45_M_Rhodoferax_ferrireducens
31_M_Herminiimonas_arsenicoxyd
76_T_Acidimicrobium_ferrooxida
75_T_Acidimicrobium_ferrooxida
34_M_Leptospira_interrogans_se
9_H_Thermobaculum_terrenum_ATC
2_A_Caldivirga_maquilingensis
98_T_Roseiflexus_sp_RS-1.fna.r
97_T_Roseiflexus_castenholzii
81_T_Chloroflexus_aurantiacus
80_T_Chloroflexus_aggregans_DS
3_A_Desulfurococcus_kamchatken
1_A_Acidianus_hospitalis_W1.gb
